# “Using dried blood spots beyond newborn screening – is Hong Kong ready?”: navigating the intersection of innovation readiness, privacy concerns, and Chinese parenting culture

**DOI:** 10.1186/s12889-024-20365-4

**Published:** 2024-10-26

**Authors:** Olivia Miu Yung Ngan, Cheuk Wing Fung, Mei Kwun Kwok, Eric Kin Cheong Yau, Shing Yan Robert Lee, Ho-Ming Luk, Kiran Moti Belaramani

**Affiliations:** 1 Medical Ethics and Humanities Unit, School of Clinical Medicine, LKS Faculty of Medicine, University of Hong Kong, Hong Kong SAR, China; 2 Centre for Medical Ethics and Law, Faculty of Law and LKS Faculty of Medicine, University of Hong Kong, Hong Kong SAR, China; 3 Metabolic Medicine Unit, Department of Paediatrics and Adolescent Medicine, Hong Kong Children’s Hospital, Hong Kong SAR, China; 4 Department of Paediatrics and Adolescent Medicine, Princess Margaret Hospital, Hong Kong SAR, China; 5 Department of Paediatrics and Adolescent Medicine, Pamela Youde Nethersole Eastern Hospital, Hong Kong SAR, China; 6 Department of Clinical Genetics, Hong Kong Children’s Hospital, Hong Kong SAR, China

**Keywords:** Newborn screening, Inborn errors of metabolism, Inherited metabolic disorders, Dried blood spots, Biobank, Hong Kong

## Abstract

**Background:**

Newborn screening programmes offer an opportunity to obtain dried blood spots (DBS) cards that contain a wealth of biological information that can be stored for long periods and have potential benefits for research and quality assurance. However, the storage and secondary uses of DBS cards pose numerous ethical, clinical, and social challenges. Empirical research exploring public attitudes is central to public policy planning as it can indicate whether or not there is broad public support, define public concerns, and ascertain the circumstances required to alleviate concerns and ensure support. This study aims to describe the clinical experience and attitudes towards newborn screening and investigate the perceptions and expectations of Hong Kong parents and healthcare providers regarding the retention of DBS cards and their usage for research.

**Methods:**

We conducted semi-structured in-person interviews with 20 parents and healthcare providers in Hong Kong. Thematic analysis was conducted.

**Results:**

Awareness of the significant research value of secondary uses of dried blood spot cards is low. Parents and healthcare providers support the storage and secondary uses of DBS cards with some concerns, including privacy and confidentiality breaches, the risk of discrimination or stigmatisation based on genetic information, and their inability to oversee the use of their child’s biospecimen. Parents, however, prioritise their child’s health over privacy concerns and support identifiable storage using pseudonymity to gain more information about their children's health.

**Conclusion:**

Child information takes precedence over potential concerns over privacy, underscoring the significance of engaging patients and the public in shaping public policy related to biobanking and healthcare research, in line with cultural and social values.

**Supplementary Information:**

The online version contains supplementary material available at 10.1186/s12889-024-20365-4.

## Background

The development of high-resolution tandem mass spectrometry has brought about a significant change in the field of newborn screening (NBS) based on Dried Blood Spots (DBS) cards [1]. This method enables the detection of inherited metabolic disorders (IMD) and numerous uncommon diseases. The DBS cards can be preserved for extended periods and have minimal risk of bacterial contamination or haemolysis [2]. Storing DBS cards provides a wealth of biological data and presents an excellent opportunity for secondary analytic purposes, including programme development and evaluation [3], public health epidemiological studies [4], and identification of missing or deceased children [5]. The secondary uses of DBS cards offer significant value in research and can benefit the population as a whole. For example, they can be used to develop screening tests for new conditions [6], forensic toxicology examinations [7], as well as to study environmental epigenetics [8]. However, the benefits to individual donors may be less apparent.

Empirical research found that parents’ knowledge of NBS and the use of DBS cards is often limited. Studies have focused on parental understanding of DBS cards obtained through NBS programs and have found that parents of young children often need more knowledge of the program and are not familiar with the handling of the specimen [9, 10]. Some parents may need to be made aware of the potential health conditions that can be detected through these tests and even be reminded of the storage policy, especially since some parents assume that all samples are discarded immediately after clinical use [11]. Despite this lack of knowledge, parents generally express high trust and are often willing to archive their child’s DBS cards if consent is obtained [12, 13]. Other studies suggest that parents may have concerns about the storage and use of DBS cards and may benefit from more information about the consent process and the potential uses of the samples [14, 15].

Assessing cultural attitudes towards biospecimen handling is paramount as it offers significant insights into communities’ beliefs, values, and opinions regarding utilising biospecimens. In New Zealand, a research team conducted a focus group study comprising 37 participants from diverse ethnic backgrounds, which revealed cultural considerations that are particularly relevant to the Mäori and Pacific people [16]. These communities view blood as sacred, and blood, including the blood on DBS cards, is believed to be part of the person they were taken from. As such, the bodies of deceased individuals should be buried whole, with all organs and blood. One notable event to demonstrate mistrust occurred in 1997 when West Australian Police secured a court order to access and analyse DNA from DBS cards as part of an investigation into a case of alleged incest [17]. This incident received national media attention and sparked public outcry, ultimately leading to the destruction of all DBS cards older than two years. These cultural beliefs and concerns have implications for developing guidelines for future uses (including research), storing, and retrieving DBS cards. Disregarding these cultural views may lead to mistrust, exploitation, and discrimination. Such actions may weaken the integrity and validity of biospecimen research, impeding progress in scientific discovery.

The existing literature on the subject is predominantly centred on Western cultures, political contexts, and social theories. However, these perspectives may not fully apply to non-Western practices due to differences in cultural ethos and moral sensibilities. While local elements of healthcare systems are paramount to developing integrated care, rigorous research has yet to be conducted in Hong Kong on how local needs and social-cultural contexts shape the healthcare experiences of parents with and without a child with uncommon disease. This is the first qualitative study exploring the clinical experience of uncommon disease screening among clinical service users and providers. The study is part of a larger project that examines the clinical and public health implications of DBS card storage and its usage for secondary uses among parents and healthcare providers (HCPs) in Hong Kong. We present the findings from the second qualitative phase, which aims to describe the clinical experience and attitudes towards newborn screening and investigate the perceptions and expectations of Hong Kong parents and healthcare providers regarding the retention of DBS cards and their usage for research.

## Methods

Using a sequential explanatory mixed-methods design, the first phase of a cross-sectional survey assessed knowledge, attitudes and practices towards the storage and secondary use of DBS cards among parents and HCPs in Hong Kong [18]. We reported that parents had low awareness and inadequate knowledge of DBS card storage. We postulated that NBS information has not been fully disseminated into primary public mediums since the NBS has only been established across all public hospitals with maternity services within the past 5 years and thus requires additional time to improve. The finding addressed the importance of implementing system-level public education and promotion interventions.

### Study context

NBS has been a part of Hong Kong's healthcare system since 1984, providing screening for congenital hypothyroidism and glucose-6-phosphate dehydrogenase (G6PD) deficiency on cord blood at no cost for babies born in public hospitals with maternity services [19]. In 2007, NBS for congenital hearing loss was added [20]. After a successful pilot in 2017 [19], a NBS for IMD using tandem mass spectrometry, covering 25 IMDs and one endocrinopathy (congenital adrenal hyperplasia), was extended at no cost for babies born in birthing hospitals within the public healthcare system in phases [21], with completion in 2020. Severe combined immunodeficiency was added to the NBS program in 2023, and spinal muscular atrophy is currently undergoing pilot testing in Hong Kong. NBS is free for babies born in public hospitals with maternity services.

### Sampling and recruitment

The target population for this study is parents who are caring for a child with or without a health condition, as well as HCPs, including doctors, nurses, dieticians, laboratory technicians, and those in other fields relating to clinical services involved in NBS.

Parents were recruited while attending the paediatric service outpatient clinic at three public hospitals. The researcher ensured that parents were fully informed about the study, and the study information leaflet included basic background information on the concepts of the DBS card and its potential during recruitment. After obtaining written consent, parents received a hard copy or an e-copy of the survey and were given an option to leave their contact information for the interview. HCPs were invited to participate in the study using snowball sampling.

Among the 452 parents and 107 HCPs who completed and returned the survey, 79 (17.5%) parents and 12 (11.2%) HCPs consented to be contacted for the interview. The interviewees were selected from the survey respondents who left their contact information for a follow-up interview. To ensure that there was no selection bias for the qualitative study, we checked for demographic differences between those who agreed to be contacted and those who did not. Our findings showed no significant patterns suggesting little concern for selection bias.

A quota-sampling matrix was constructed to invite participants for interviews. For parents, the selection was based on age, education, child’s condition, and household income. For HCPs, the selection was based on profession, speciality, and role in NBS. Simultaneous data collection and analysis enabled the identification of emerging themes from the outset. As the interview progressed, snowball sampling was adopted to recruit HCPs based on the emerging themes. We invited obstetric providers based on the study objectives and demographics, aiming to maximise the variability of the sample to reach saturation (i.e., obtain a comprehensive understanding until no new information is acquired). We reached data saturation of the key themes around the seventh interview and decided to complete the tenth interview to ensure the inclusion of participants according to our sampling matrix. Written informed consent was obtained from all study participants, and their participation was entirely voluntary. In-depth interviews were conducted in their home, coffee shops, or offices, lasting 30 to 75 min, and all were completed between December 2022 and April 2023.

### Interview guide

We subsequently developed a semi-structured in-depth interview guide to qualitatively explore the findings from the quantitative phase and discrepant views and attitudes towards DBS card storage (see Supplementary Material 1). The interview domain broadly explored clinical experience and attitudes towards newborn screening, and retention, storage, and use of DBS cards. This included understanding towards DBS cards, informed consent approach, opt-in vs opt-out method, length of storage, and potential clinical and ethical concerns. The interview guide was piloted with a nurse and two parents to ensure content validity, relevance, and clarity. These data were not included in the data analysis.

### Data analysis

The interviews were conducted in the language preferred by the participants. Audio recordings of the interviews were transcribed verbatim and translated into Chinese by an independent, bilingual researcher to ensure the accuracy of the translations. The quotes were subsequently translated into English for publication. Thematic narrative analysis was used to identify and analyse key themes from the interviews. A constant comparative method of coding was used to create the themes. The analysis focused on explanatory investigations to better understand interviewees’ knowledge of uncommon diseases and DBS cards, as well as their opinions on storing DBS cards for secondary usage. The themes were then reviewed and refined by the research team to ensure consistency and accuracy of interpretation. To increase the credibility and transferability of the findings, member checking with experts was conducted, and their feedback was incorporated into the final analysis.

## Results

### Demographics characteristic of interviewees

Table 1 describes demographics characteristic of interviewee. A total of 20 interviews were conducted among ten parents and ten HCPs. The majority of parents were female (90%) and were sampled from age groups: one aged 21–25 (10%), one aged 26–30 (10%), four aged 31–35 (40%), three from 36–40 (30%), and one from 41–45 (10%). Their highest level of education ranged from an associated degree or lower (50%), and tertiary education or above (50%). There were 8 (80%) parents who were taking care of a child with a disease, of whom 4 (40%) were suffering from IMD and uncommon disease, 1 (10.0%) from cardiac disease, 1 (10.0%) Temple syndrome, 1 (10.0%) from vascular nevus, 1 (10.0%) from undiagnosed uncommon disease related to muscle. No parents share the same child.

**Table 1:** Demographics of interviewees

	**Parents *n* = 10**		**HCPs *n* = 10**	
	n	(%)	n	(%)
**Gender**				
Female	9	(90%)	9	(90%)
Male	1	(10%)	1	(10%)
**Age**				
21–25	1	(10%)	-	-
26–30	1	(10%)	1	(10%)
31–35	4	(40%)	3	(30%)
36–40	3	(30%)	2	(20%)
41–45	1	(10%)	4	(40%)
**Education**				
Associate degree or lower	5	(50%)	-	-
Tertiary or above	5	(50%)	10	(100%)
**Child’s diagnosed Conditions**				
None	2	(20%)	-	-
Inherited Metabolic Disease / Uncommon Disease	4	(40%)	-	-
Temple Syndrome	1	(10%)	-	-
Vascular Nevus	1	(10%)	-	-
Undiagnosed rare disease related to muscle	1	(10%)	-	-
Cardiac Disease (Patent ductus arteriosus)	1	(10%)	-	-
**Position**				
Nurse	-	-	8	(80%)
Doctor	-	-	2	(20%)
**Field**				
Obstetrics and Gynaecology	-	-	2	(20%)
Paedeatrics (Neonatal Intensive Care Unit, Post-natal Ward)	-	-	7	(70%)
Chemical Pathology	-	-	1	(10%)
**Role in the Newborn Screening**^**a**^				
Introduce Programme and Obtain Consent	-	-	2	(20%)
Disclose Results to Parents	-	-	2	(20%)
Blood Taking	-	-	5	(50%)
Provide Education to the Department	-	-	1	(10%)
Lab Testing	-	-	1	(10%)

^*a*^*Some involved more than one role and therefore sum up more than 100%*

HCPs respondents were female (90%), nurses (80%), work in the paediatrics department (70%), and aged between 26–30 (10%), 31–35 (30%), 36–40 (20%), and 41–45 (40%). All obtained tertiary education or above. Their involvement in newborn screening was obtaining consent (20%), disclosing test results to parents (20%), conducting laboratory tests (10%), conducting an educational seminar (10%), and drawing blood (50%).

### Clinical experience of newborn screening

#### Attitude towards newborn screening

The diffusion of NBS knowledge varies. There were seven parents who had a child with a condition, but not all were diagnosed through NBS. Some had conditions like temple syndrome, which was diagnosed clinically.

Among ten parents, four parents had not taken part in NBS. Parents’ knowledge about the conditions being screened was driven mainly by word of mouth among friends, first-hand experience with uncommon disease, and scientific background. They recognised that a delay in diagnosis would lead to a detrimental effect on the child’s health. Parents were willing to pay for the test out of their pocket even before it was available at public hospitals with maternity services at no cost.

*A friend of mine had a child who was diagnosed with a metabolic disease during second-tier screening. The child displayed significant speech and motor skills delay and received extensive training. Since some diseases are rare, it is essential to participate in screening programs to detect such conditions as early as possible. Even though newborn screening for uncommon disease was not covered for free during my pregnancy, it was still money well spent on early screening. (Parents #3, child with vascular nevus)*

Parents demonstrated an understanding of why the screening was performed and that screening would allow for early intervention if a condition were identified. They cited that the benefits of receiving more information about a child outweigh the risks of a heel prick test, also commonly called “just a poke” test. It has a parallel meaning of minimal risk. In particular, one parent was touched by recent news coverage about a paediatric patient suffering from a rare oncology condition, raising her empathetic awareness of prioritising a child’s health at all times.

*My child's health comes first. My heart was heavy when I saw a recent news about the demise of a child who had been diagnosed with a rare kind of cancer called neuroblastoma, and another pediatric patient who was widely known across the territory to have a congenital heart defect and had undergone at least six surgeries by the age of four. (Parent#2, child has no disease)*

Similarly, HCPs agreed that the inception of universal uncommon disease screening had been shown to significantly increase the detection rate of uncommon diseases, allowing for earlier interventions and improved management of these conditions. Before the implementation of this programme, however, there were documented cases of IMD that presented as sudden decompensation and required urgent medical intervention. In this context, a paediatrician emphasised the pivotal role of the DBS card-based NBS programme in preventing such incidents.

*There are documented cases of methylmalonic acidemia and carnitine acylcarnitine translocase deficiency in medical literature. These cases exhibited rapid decompensation, with one case necessitating hemodialysis and the other requiring resuscitation. The implementation of the DBS card-based NBS program could have prevented these incidents. (Doctor #9, Paediatrics)*

During a decade of clinical practice, another nurse came across a unique case illustrating the potentially detrimental consequences of delayed or missed diagnosis of uncommon disease. Their experience highlights the importance of early detection. While these disorders may be individually rare, collectively, they affect a significant number of newborns worldwide.

*The child's health rapidly deteriorated, leaving no immediate targeted treatment options. Oxygen therapy was the only available intervention to manage episodes of desaturation. Eventually, a diagnosis of an enzyme deficiency was established, emphasising the importance of timely diagnosis to improve health outcomes and reduce suffering. Parents became very frustrated when they saw their child deteriorate quickly. (Nurse #5, OG)*

#### Consent procedures for newborn screening

NBS requires collaboration between the Obstetrics and Gynecology and Paediatrics departments. HCPs from the antenatal team discussed their respective roles in providing information and obtaining consent from pregnant individuals and families.

*As part of our prenatal care protocol, we provide expectant parents information about the newborn screening program at approximately 24-27 weeks. The information is delivered through a combination of video and pamphlets. After a verbal consultation with our colleagues, parents are requested to provide written consent by signing a paper form. This form is then documented in the parents' medical records. If parents declined participation in the newborn screening program, this decision is also documented. Importantly, we inform parents that they can change their decision at any time up to 72 hours after delivery. (Nurse#10, OG)*

At the neonatal unit, HCPs review the documentation of the consent form and parents’ medical records before conducting the heel prick test.

*The neonatal unit does not provide detailed pre-test counselling like the antenatal unit. Instead, we act as facilitators in blood taking, confirming parents’ consent and ensuring their understanding of NBS. Sometimes, when consent is not given initially, or the mother cannot recall the newborn screening for uncommon disease, we provide mothers with brief information. (Nurse#4, Paediatrics).*

Parents proactively sought information from multiple sources, such as online discussion forums and chatrooms, to gather knowledge regarding their child's health condition. However, parents may encounter difficulty comprehending the information due to their limited scientific literacy. Therefore, there is a pressing need for comprehensive public education programs that can equip parents with the necessary tools to access and understand important information related to their child's health. Such education programs would not only promote parental understanding of their child's health condition but also enhance their ability to make informed decisions regarding their child's health.

*The technical terminology in the leaflet was difficult to understand. In addition to medical knowledge, I would appreciate the pamphlet to include parents’ voices, real-life experiences, and concerns about the diseases and treatment. It helped us gain perspective as prospective parents. (Parent#10, child with temple syndrome)*

When asked about their recollection of newborn screening, parents often responded with self-deprecating remarks, claiming that they didn’t remember anything. Many attributed this to the confused nature of the post-birth period, particularly in an unfamiliar hospital setting with new procedures. Sleep deprivation and emotional stress also limited their recall of specific details about the heel prick test.

*The 10-month pregnancy was pretty distressing. From what I recall, they handed me a consent form to conduct a series of check-ups for the newborn. I gave my signature. I was not in the mood to read the pamphlet. (Parents #8, child with a rare muscle disease)*

*They provided an information sheet including some details that were written elaborately. I had little motivation to read the content and decide whether or not to participate in the screening. However, an immediate response was much needed. (Parent#5, child with cardiac disease)*

A number of nurses were not surprised to learn that many parents were unable to recall any information about NBS for uncommon diseases. They acknowledged that parents are often overwhelmed with information during the prenatal and postnatal periods and that the importance of NBS may not be immediately apparent to them. Despite this, the nurses emphasised the importance of educating parents about the benefits of NBS, which include the early detection and treatment of rare disorders.

*At the 24*^*th*^* week of gestation, parents are bombarded with information on various topics such as gestational diabetes, breastfeeding, episiotomy, newborn screening programme, and details about upcoming clinical follow-ups. Although they receive flyers on these topics, the sheer volume of the information might lead to confusion (Nurse#10, OG)*

Some HCPs have questioned the need for parental consent for NBS procedures. They argue that parents may lack sufficient knowledge or understanding of the process, making it difficult to make an informed decision. Additionally, some HCPs believe that the consent process may appear forced and unnecessary. They debated the possibility of incorporating screening for uncommon disease as mandatory screening, similar to G6PD deficiency and thyroid screening already in place for all newborns.

*Newborns at the ward took blood tests frequently, like every 4-8 hours. Mothers rarely asked us about the purpose of the blood collection, “What’s that for? When will we get back the results ?” It was just another normal procedure at the ward. (Nurse#1, Paediatrics)*

### Storage and secondary usage of the DBS cards

#### Awareness

While nearly all parents recalled the heel prick test, none could remember receiving information about storage and future use. Two parents were either unfamiliar with the test procedure or unaware of the card’s existence. All parents were surprised to learn that blood spot cards could be stored and had great research potential.

*I saw the nurse take five drops of blood on the card, which I thought would be discarded after the screening test. I did not think it could be reused. I assumed it would only last a few days. (Parent#1, child diagnosed with an IMD)*

*As mothers, we only want to safeguard our child’s health; other things do not matter. Also, I was tired after giving birth. I had no idea about the exact screening procedure or the card. (Parent#8, child with a rare muscle disease )*

Many HCPs involved in NBS were found to lack awareness regarding the secondary uses of the DBS cards, including for research purposes.

*I believed that the DBS card could only be used for screening and nothing else. I did not know it could be kept for future use, and I had no idea how long it could be stored. (Nurse#2, Paediatrics)*

#### Drivers to support secondary usage

Interviewers described the potential of DBS cards for various research purposes apart from NBS, including diagnosis and disease surveillance. These DBS cards can be stored for long periods, sometimes decades, and can be used for purposes that could benefit the general population and occasionally on an individual level. After learning about the utility of DBS cards, parents unanimously support secondary usage and storage for several reasons. Firstly, storing DBS cards provides a valuable resource for future research, which can be beneficial for public health. By having an extensive collection of biospecimens, researchers can study the prevalence of diseases and conditions in different populations, which can inform public health policies and interventions. Although their child would not directly benefit from the storage, parents discussed the altruistic beneficence to society.

*A single instance of providing consent is deemed adequate for the purpose of research, which aims to enhance medical understanding for the benefit of humanity. (Parent#10, child with temple syndrome)*

It gives parents a sense of satisfaction that they, too, can contribute to advancing medical knowledge and improving health outcomes for future generations. In addition, storing DBS cards ‌can give parents a sense of reassurance, knowing that their child’s biospecimen is securely and ethically stored for future clinical or research purposes. They hope that any abnormal results found through research will benefit their child through reciprocal participation.

*My child was diagnosed with an IMD condition. I did not understand or care about how the card would be handled as long as my participation in the secondary storage and research motives reaffirms my child’s health. Many diseases are not included in the screening panel. I was happy to consent to use the residual blood for further analysis if it can generate other information about my child. (Parent#1, child with an IMD)*

*I place greater importance on health than privacy. In the case of an uncommon disease diagnosis for my child, anonymous storage would not be helpful. If the DBS cards‌ are stored with identifiable information, a technician could contact me immediately without any delay. Therefore, I support connecting health information with the card ‌. (Parent#7, child with an uncommon disease)*

Storing DBS cards can be an ideal candidate for biobanking. By using identifiable information, researchers can conduct more comprehensive and accurate research. Linking the biospecimens to health information can help researchers better understand the factors contributing to disease, develop more effective treatments and preventative measures, and, more importantly, ultimately benefit children.

*I would be concerned that it could lead to breaches of personal data. However, I would agree if the card is used to discover some unknown diseases (personal uses - my child might have some hidden / undiagnosed disease that could be discovered one day). (Parent#1, child with an IMD)*

When it comes to conducting research, having samples available is incredibly advantageous because it allows for secondary analysis to be performed without the need to call back patients for another round of blood collection. This not only saves time and resources but also reduces the burden on patients who would otherwise need to provide additional samples for research purposes. Moreover, samples can be used to study numerous medical conditions, making them a versatile resource for researchers.

*Having samples available for research would be helpful because scientists could study them without having to bother patients to travel to hospitals for more blood tests. (Nurse#1, Paediatrics)*

#### Concerns

Parents who receive an abnormal NBS result or an early diagnosis, experience a mix of emotions, including gratitude for the information that could improve their child’s health and development, as well as a sense of loss due to the unexpected diagnosis that irrevocably alters the process of bonding with their child during the initial weeks or months of parenthood. Similarly, other parents who have a child with an uncommon disease may experience a range of emotions, such as disbelief, frustration, guilt, and anxiety. Since the diseases screened are uncommon, it can be challenging for parents to find accurate information about the condition and its treatment options. Caring for a child with an uncommon disease can take an emotional toll on parents.

*I felt depressed and frustrated because I did not understand the disease. She was my first child. Unfortunately, my child’s IMD diagnosis was not included in the screening panel of the programme. My child had been diagnosed with glycogen storage disease in the first month of life with another test. (Parent#4, child with an IMD)*

A few parents expressed apprehension about using DBS ‌cards for research purposes. They recognised that DBS cards store sensitive material that requires dedicated handling and careful planning in data management. They worry about several issues, such as the possible loss of privacy and confidentiality, the risk of discrimination or stigmatisation based on genetic information, and their inability to oversee the use of their child’s biospecimen. Additionally, they worry about the potential misuse of their child’s biospecimen, including its usage for commercial purposes or in research that contradicts their values or beliefs.

To tackle privacy concerns, maintaining anonymity is a feasible solution to support the DBS card research. This could be achieved in two ways: By ensuring complete anonymity and non-traceability or by using a consistent identifier that is not the actual name of the users, creating a near-anonymous state. Parents are well aware that the former anonymous storage may result in loss of contact.

*“I supported non-anonymous storage. The overall aim is to explore my child’s health. How can the researcher identify me? Without identifiable information, it isn’t significant. From my understanding, research usually reports aggregated data only. (Parent#10, child with temple syndrome)*

Despite privacy concerns, some parents chose to prioritise health over privacy concerns regarding the secondary uses of DBS cards for research because they believe that the benefits of the research outweigh the risks. They think that identified storage could aid future research and benefit their children. Therefore, they are not overly concerned about privacy. This could result in the creation of novel diagnostic tools and treatments, which could enhance health outcomes for individuals and communities both.

*When it comes to my child’s health, I prioritise it over privacy. As long as researchers adhere to privacy guidelines, privacy concerns are implausible. (Parent#9, child with an IMD)*

HCPs favour the second near-anonymous state approach in which only a few research members would have access to the identity.

*If we want to help the patient, we can keep their card and identity anonymous. That would be helpful for their parents. But we can add a code or ID that only trusted institutions could use to identify them. This way, researchers would not be able to know who the person is directly. (Nurse#7, Paediatrics)*

Furthermore, parents are concerned about the informed consent procedure and whether they completely understand the consequences of utilising their child’s biospecimen for research. They also worry about how biospecimen research could affect their child’s future healthcare or insurance coverage.

*Blood contains genes that can be extracted. Although it might not be used for any practical purposes, it can be used to diagnose diseases. Some have not been developed, which may affect our privacy in the future - personality, defects, and things we do not necessarily want to know. People do not want insurance to know much. (Parent#9, child with an IMD)*

Parents with experience with a particular health condition or disease are mainly motivated to participate in biospecimen research that could lead to better treatments or cures. Some reflect on the feasibility of privacy in the modern digitalised world. Parents question the extent of privacy in the modern digital age, where we are forced to surrender our data in many ways.

*Research is a way to find new diseases and treatments to benefit people in need; it is good. Privacy is not entirely upheld in the contemporary digitalised age. Of course, there are many conspiracies that a researcher can use a particular person’s blood for a hidden project. I support research (Parent#7, child with an uncommon disease).*

#### Length of storage

The length of time that parents support storing DBS cards may vary depending on several factors. Some parents are supportive of storing DBS cards indefinitely. One factor influencing the length of time parents support storing DBS cards is their perception of biospecimen research’s potential benefits and risks. If parents believe that the benefits of storing DBS for future research outweigh the risks, they are more supportive of long-term storage.

*“Child’s health has much uncertainty (e.g. undiagnosed disease) up until 3 years of age. I support storage if technologies could use the stored DBS cards for further analysis that helps with other diseases, early intervention and treatment. The database is fundamental to support analytic development.” (Parent#7, child with an uncommon disease)*

Another factor influencing the length of time parents support storing DBS cards is the type of research to be conducted. Most parents were comfortable with the idea of indefinite storage for analytic assurance or research on a particular health condition or disease that affects their child. In particular, parents taking care of a child diagnosed with a condition that requires long-term medical care fully support extending the storing of the DBS cards for research purposes.

*“Six months is too short. Most children with uncommon disease develop symptoms after six months of age, like when the child started to look up or starts to walk. It is hard to determine whether development is normal between 0 and 6 months. I support indefinite storage provided that facilities can support it. (Parent#8, child with a rare muscle disease).”*

## Discussion

The study is the first to investigate the attitude and knowledge of NBS for uncommon disease following its implementation in birthing units within the public healthcare system in 2020 [18]. The study findings revealed that parents and HCPs perceive NBS screening for uncommon disease as a significant public health milestone. In particular, HCPs discussed that the opt-out model is preferable to the current opt-in model. The primary rationale for this is driven by simplifying the screening process and, secondly, preventing missed diagnoses of treatable conditions, which can lead to dire outcomes [22, 23]. A team in the United States leading a pilot screening program discussed the dangers of parental refusal to have their infants screened for serious or treatable disorders included in an NBS panel. Such refusals, whether due to lack of knowledge, can result in severe disability or even death [24]. Although the opt-out approach has elements of paternalism, it is not uncommon. A review focusing on European regions found that most countries mandate NBS, with participation rates exceeding 98% [1]. The associated benefits include ensuring uniform screening for all newborns, regardless of socioeconomic background or healthcare accessibility, promoting healthcare equity, and reducing health disparities. However, there are conflicting opinions among parents, as some explicitly express their opposition to mandatory screening and feel deprived of choice [25]. Ross et al. contended that while some medical professionals assert that parents possess an absolute right to make decisions about their children’s health, this right should be exercised judiciously within the bounds of evidence-based care and without neglect or abuse. [26] In a similar vein, Ulph et al. emphasise the critical importance of informed consent. They underscore that parents should receive comprehensive information about the screening process and fully understand that their consent covers both the screening itself and the subsequent storage of bloodspot samples. [27].

In the context of Hong Kong, where participation rates for the opt-in approach currently exceed 98% [28, 29], considering an alternative opt-out approach could be a feasible option. This alternative approach would streamline the screening process and address parental refusal concerns while respecting the parents' right to opt-out in accordance with their beliefs. The primary rationale behind the initial implementation of the opt-in explicit consent-based approach was the involvement of a heel prick procedure. However, our study finds that local parents do not appear to be too concerned about this procedure. Exploring an opt-out approach, accompanied by a comprehensive cost-effectiveness analysis, becomes imperative to justify allocating limited healthcare resources to this endeavour and inform public policy decisions. While Hong Kong’s current opt-in approach remains acceptable, given parental consent preferences, it may be more fitting to pursue bio-banking or extended genetic testing. However, we should critically reevaluate the timing of obtaining consent and the use of medical terminology in informational materials. In an opt-out approach, we must also thoughtfully consider implementing alternative measures when incidental findings beyond the disease panel arise [30]. Moreover, cultural appropriateness plays a crucial role in determining the most suitable consent model, considering our community's diverse perspectives and values.

Similar to other studies, local parents and HCPs were not fully aware of the potential benefits of DBS cards [18, 31]. They discovered that long-term storage permits retrospective screening if a child unexpectedly develops a condition and aids in assessing the prevalence and feasibility of possible new additions to the screening panel. However, they also expressed concerns about the traceability of personal data and the challenges associated with managing long-term storage under appropriate conditions. In many regions, including Australia [32], China [33], Noway [34], and the United States [35], fundamental privacy concerns are rooted in mistrusting authorities and the medical system, and the lack of fiduciary relationships between researchers and patients has led to reluctance to biobank research participation [36, 37]. Parents are hesitant to permit the storage of their child's samples without explicit consent, advocating for a more active role in policy-making alongside researchers [12, 13].

As Hui et al. [15] and Cheung et al. [38] reported, concerns about privacy and stigmatisation in handling genetic information are not uncommon among the local population; however, local parents in our study tend to place considerable trust in the researchers involved in biospecimen research. In particular, contrary to the academic literature that privacy concerns and the anonymous handling of samples are predominant hesitations when considering participation in scientific research [39–41], many local parents prioritise their child’s health above these issues. They prefer that their child’s biological sample be stored in an identifiable manner — be it non-anonymous or pseudonymous—so it can be readily available for any future diagnostic or therapeutic needs. The choice to engage in NBS for more information reflects a broader social and cultural inclination to favour direct and discernible health benefits for children over the more nebulous concerns associated with biobanking privacy. This inclination shows that with sufficient education about the safeguards for their child’s biological information, parents are inclined to continue endorsing biobanking activities (e.g. storage of DBS cards), recognising the essential role these measures play in protecting their children's health in the long term. The local preference for child information over privacy emphasises the importance of patient and public involvement in shaping public policy for biobanking and healthcare research responsive to cultural and social values.

Concerns about the traceability of personal data and the challenges of managing long-term storage in appropriate conditions should be addressed. At present, the legal framework for protecting personal data in Hong Kong has been evolving. There is no specific legislation in Hong Kong related to establishing or operating a biobank [15]. However, there are general regulations and guidelines that apply to the processing of genetic data by third parties (i.e. other than the data subject and those authorised directly for data processing). These include the Discrimination Ordinance (Cap. 487) [42], the Personal Data (Privacy) Ordinance (Cap.486) [43], and the Hong Kong Academy of Medicine guidelines [44]. Despite the implementation of a legal framework, some local researchers [38] have noted that the laws are not comprehensive or specific enough to prevent insurance companies and employers from manipulating employees' genetic information. Disputes over genetic discrimination have occasionally been reported [38, 45]. As NBS matures, it is essential to implement specific regulatory measures to prevent genetic discrimination in life and health insurance, especially concerning health data [43]. This can influence public perception and should be carefully monitored.

## Study limitations

While the study's insights are valuable in developing patient-centred biobanking public policy, it is important to acknowledge several limitations. Firstly, the study sample was limited to a few healthcare providers involved in NBS at public hospitals with varying roles. Although the HCPs sample may appear homogenous, it is representative of the distribution of HCPs in Hong Kong, as evidenced by the 2022 census and statistics of Hong Kong, which reported a doctor-to-nurse ratio of 1:4.2, a proportion consistent with our HCPs sample [46]. Furthermore, the 2019 Health Manpower Survey on Registered Nurses, conducted by the Department of Health Hong Kong, reported that 86.7% of registered nurses were female, a proportion similar to our sample [47]. Notwithstanding these limitations, the study yielded valuable insights into the experiences of healthcare professionals involved in newborn screening and will inform future research in this area.

Secondly, the study's parent participants were residents of the local area with diverse demographic backgrounds and parenting experiences, including those with and without children affected by diseases. Their experiences may influence their views on biobanking policy. Lastly, parents were selected based on diverse demographic backgrounds and parenting experiences, including those with and without children affected by diseases. The unique experiences of parents who have dealt with diseases in their children may provide valuable insights into the potential benefits and concerns surrounding biobanking and its impact on healthcare research. However, these findings should be interpreted carefully. Further research with a larger and more diverse sample is needed to confirm and expand upon these findings.

## Supplementary Information

Supplementary Material 1.

## Data Availability

Data is available upon reasonable request from the corresponding author.
