# Supplementary material for: “Using dried blood spots beyond newborn screening – is Hong Kong ready?”: navigating the intersection of innovation readiness, privacy concerns, and Chinese parenting culture
Source: BMC Public Health. 2024 Oct 26;24:2973. doi: 10.1186/s12889-024-20365-4 (PMC11515137; doi:10.1186/s12889-024-20365-4)
Supplement: Supplementary file 1 — Supplementary Material 1. [file 12889_2024_20365_MOESM1_ESM.docx]

**Semi-structured Interview Guide**

| 1. **Clinical Experience with Newborn Screening (*Parents Only)** |
| --- |

1. Have you participated in the Newborn Screening for uncommon diseases?
2. Please describe your experience of Newborn Screening.
   - Where did you receive the information about the screening programme?
   - Where did you receive the service?

1. Have you heard about Dried Blood Card Storage (DBS Card)?
   - If yes, when and where did you receive the information?
   - Who deliver you the information?
   - What do you think about the quality of information?
2. Have you stored your child’s dried blood card?

1. **Clinical Experience with Newborn Screening (*Healthcare Provider Only)**
2. Have you involved in the Newborn Screening for uncommon diseases?
3. Please describe your clinical experience of in offering Newborn Screening.
4. Have you heard about Dried Blood Card Storage (DBS Card)?
   - If yes, when and where did you receive the information?
   - Who deliver you the information?
   - What do you think about the quality of information?

| 1. **Attitude towards Storage of Dried Blood Spot Card (DBS card) and its future use** |
| --- |

Present the Vignette from the Survey.

*“Following the birth of the infant, a health professional will perform a heel prick to collect a few drops of blood on a special card. This card will be sent to the laboratory to screen for rare, but serious conditions that can be treated early for the long-term benefit of affected babies. Moreover, the information obtained from this screening has the potential to benefit the paediatric population and further scientific research. Currently, the card is retained for six months after the test has been performed for quality assurance purposes, after which it is discarded. In contrast, other countries may retain the card for several years or even decades for the purposes of conducting further research.”*

1. Currently, the DBS card will only be stored for 6 months for quality assurance purposes. Do you know what other usages the card can offer?
2. Storage of DBS card offer much promising prospect in paediatric clinical services and research. What are your view towards the below usage?
   - Public health?
   - Individual health: child or family?
   - Pharmaceutical company vs. University?
   - Victim identification?
3. DBS cards offer other opportunities for screening development and research. Are you willing to contribute the DBS card for research? What are the facilitators? barriers?
4. How long is a reasonable timeframe to be stored?

| 1. **Attitude towards Usage for Extended Genetic Testing** |
| --- |

1. At present, some countries, such as UK and US, are screening newborns using advanced technology i.e. extended genetic sequencing. The aim is to probe the risks and benefits of sequencing newborns' genome to screen for disorders before they present. For example: Baby has at least two inherited mutations implicated in heart disease but no signs of illness in the parents or child. Another baby can have mutations that can cause an enzyme deficiency; and although asymptomatic at the moment, the baby can be regularly monitored and treatment can start as soon as disease presents.
   - What are your views on extended genetic tests?
   - What are the benefits?
   - What are your concerns?
2. Also, mutation genes of hereditary diseases, such as the BRCA2 gene, which is linked to breast cancer, can be found too.
   - What are your views?
   - Would you disclose this info to your family / patients?
   - Would you hide or disclose this info to your child / patients?
   - When would you decide to disclose?
   - Concerns?

**Debrief**

*This concludes the spoken part of the interview. Before we end, I’d like to know your opinion on our interview.*

1. Do you have any questions about the interview or the study?
2. How was the interview experience for you? Was there anything that made you uncomfortable or feel offended in any way? *[If so, apologize, ask for specific feedback. Tell the respondent that you will bring back this feedback to the research team.]*

Thank you for your participation in this interview. We appreciate the time you took to talk with us. If you have more questions or comments about the study, feel free to contact the Principal Investigator whose phone number is included in the copy of the Information Sheet I provided you with.
